# Salmonellosis impacts the proportions of faecal microbial populations in domestic cats fed 1–3-d-old chicks*

**DOI:** 10.1017/jns.2014.20

**Published:** 2014-09-25

**Authors:** K. R. Kerr, S. E. Dowd, K. S. Swanson

**Affiliations:** 1 Department of Animal Sciences and Division of Nutritional Sciences, University of Illinois at Urbana-Champaign, Urbana, IL, USA; 2 MR DNA (Molecular Research LP), Shallowater, TX, USA

**Keywords:** Feline nutrition, Raw diet, Salmonella

## Abstract

There has been a recent increase in the feeding of unconventional diets, including whole-prey diets, to domestic pet cats. Our objective was to characterise faecal microbial populations of domestic cats fed whole and ground (6·35 mm grind) raw 1–3-d-old chicks (Rodent Pro). Faecal samples were collected from neutered male domestic cats (mean age = 5·7 years) fed these diet items in a crossover design. Bacterial DNA was isolated from faecal samples and amplicons of the 16S rRNA V4–V6 region were generated and analysed by 454 pyrosequencing. Faecal microbial populations of cats fed whole *v.* ground chicks did not differ. During the study, three cats presented with symptoms of infection (anorexia or diarrhoea) and tested clinically positive for *Salmonella* using a standard PCR method. The remaining cats tested negative. Data were analysed *post hoc* to test for differences in microbial populations due to clinical status. The predominant genera were *Clostridium* (9–30 %), unidentified Lachnospiraceae (10–28 %), *Blautia* (4–19 %), *Peptococcus* (2–19 %) and *Fusobacterium* (2–14 %). Faeces of cats testing clinically positive for *Salmonella* had higher (*P* ≤ 0·05) proportions of the genera *Coprococcus* (5·6 *v.* 0·4 %) and *Escherichia* (subgenera *Shigella*; 1·1 *v.* 0·3 %). *Salmonella* was not detected in faecal samples utilising the pyrosequencing method; however, there was a shift in microbial populations due to clinical status. The clinical symptoms reported herein may be not only due to the *Salmonella* itself, but also shifts in other gut microbial populations.

There has been a recent increase in feeding unconventional diets, including whole-prey diets, to domestic pet cats. Feeding whole prey mimics the diet of small wild cats, which typically eat small mammals, reptiles, birds and insects^(^^1^^)^. Whole-prey items fed raw require minimal processing, including no heat treatment or addition of preservatives. Dietary processing and ingredient and nutrient composition impact faecal microbial populations in cats^(^^2^^–^^8^^)^; however, little is known about the microbial population of cats fed a ‘wild-type’^(^^7^^,^^8^^)^.

Feeding raw foods may increase risk of exposure to potentially pathogenic bacteria for companion animals and human subjects. Whole-prey diet items containing gut contents make it inevitable that the animals ingesting them will be exposed to foreign bacteria, which may be pathogenic^(^^9^^–^^11^^)^. Infection of cats, dogs and human subjects with pathogenic bacteria have been linked to contaminated pet foods, including raw meat-based foods, commercial dry foods and wild-caught prey^(^^9^^,^^11^^–^^14^^)^. During infection, pathogenic bacteria compete with and displace the commensal species resulting in dysbiosis of microbial population and gastrointestinal upset^(^^15^^)^. The authors are aware of no studies that have utilised pyrosequencing to examine the faecal microbial populations of infected cats fed raw diets.

Our primary objective was to characterise the microbiota of cats fed whole and ground 1–3-d-old chicks. During the study three cats presented with symptoms of infection (anorexia or diarrhoea), and multiple pathogenic bacteria were detected in their faeces utilising PCR and culture techniques, including *Salmonella*. Therefore a secondary aim was to examine the alterations in the faecal bacterial populations of cats with symptomatic salmonellosis compared with those which tested negative for *Salmonella*.

## Experimental methods

### Study design

The animal protocol was approved by the University of Illinois Animal Care and Use Committee. Faecal samples were collected from neutered male domestic cats (*n* 12; mean age = 5·7 years; body condition score 4·5–5·5 of 9). A crossover design was utilised to test the impacts of two dietary treatments: (1) whole or (2) ground (6·35 mm grind) 1–3-d-old chicks (DM: 25 %, crude protein: 71 % DM basis, fat: 20 % DM basis; Rodent Pro). Fresh water was available *ad libitum*. Cats were adapted to diets for 10 d prior to fresh faecal collection (<15 min from defection). Faecal samples were stored at −80°C until DNA extraction.

### Sample analysis

Faecal bacterial DNA was isolated according to procedures described previously^(^^16^^)^ using the MO BIO PowerSoil™ Kit (MO BIO Laboratories, Carlsbad, CA, USA). Amplification of a 600 bp sequence of the V4–V6 variable region of the 16S rRNA gene was done using barcoded primers as previously described^(^^17^^)^. PCR amplicons were further purified utilising AMPure XP beads (Beckman-Coulter Inc.). Amplicons were combined in equimolar ratios to create a DNA pool that was used for pyrosequencing. DNA quality of amplicon pools was assessed before pyrosequencing using a 2100 Bioanalyser (Agilent Technologies). Pyrosequencing was performed at the W. M. Keck Center for Biotechnology at the University of Illinois utilising a 454 Genome Sequencer and FLX titanium reagents (Roche Applied Science).

### Data analysis

High-quality (quality value > 25) sequence data derived from the sequencing process was processed using a proprietary analysis pipeline (www.mrdnalab.com) and as described previously^(^^18^^)^.

### Clinical testing

During the study, three cats presented with symptoms of infection (anorexia or diarrhoea). Two diet samples and faecal samples for cats with (*n* 3) and without (*n* 9) symptoms were submitted to the Veterinary Diagnostic Laboratory (University of Illinois) for *Salmonella* detection using PCR, *Salmonella* antimicrobial resistance (*n* 3 positive samples only), *Campylobacter* detection using PCR, aerobic culture and anaerobic culture. Positive detection of *Salmonella* was verified by isolation and culture. The cat that presented with anorexia was removed from the larger study. A faecal sample had already been obtained prior to his symptoms manifesting.

### Statistical analysis

Sequence percentages at each taxonomic level were analysed using the Mixed models procedure of SAS (version 9.3; SAS Institute, Cary, NC, USA). The fixed effect of diet was tested. Means were separated for treatments using a Fisher-protected least significant difference with Tukey's adjustment. Results are reported as least-squares means with *P* ≤ 0·05 defined as significant and *P* ≤ 0·10 as trends for treatment effects. Genera data and select species data were analysed *post hoc* to test for differences in microbial populations due to clinical *Salmonella* status.

## Results

Dietary treatment did not impact proportions of bacterial phyla or genera (data not shown). Firmicutes (62–86 % of all sequences) was the predominant bacterial phylum in cat faeces. Proteobacteria (0·6–16 % of all sequences), Fusobacteria (2–14 % of all sequences), Actinobaceria (1–10 % of all sequences), Tenericutes (0·7–9 % of all sequences) and Bacteroidetes (0–0·7 % of all sequences) were also present (data not shown). The predominant genera were *Clostridium* (9–30 % of sequences), unidentified Lachnospiraceae (10–27 % of sequences), *Blautia* (4–19 % of sequences), *Peptococcus* (2–9 % of sequences) and *Fusobacterium* (2–14 % of sequences).

The three cats exhibiting symptoms of infection (anorexia or diarrhoea) tested clinically positive for a group B *Salmonella* species using a standard PCR assay (Table 1). Diet samples and the remaining cats tested negative. The *Salmonella* was resistant to the antibiotics Clindamycin, Erythromycin, Penicillin and Oxacillin, and susceptible to Amoxicillin, Ampicillin and fourteen other antibiotics. Regardless of symptoms, the results were similar for *Campylobacter*, aerobic culture and anaerobic culture. *Salmonella* was not detected in any of the samples utilising pyrosequencing; however, the proportions of select faecal genera were modified in cats with symptomatic salmonellosis (Table 2). Cats with symptomatic salmonellosis had increased (*P* ≤ 0·05) proportions of *Coprococcus* and *Escherichia* subgenera *Shigella*, and tended to have decreased (*P* ≤ 0·09) proportions of *Oscillobacter* and *Anaerotruncus*. Twenty-eight species from the genera *Campylobacter*, *Clostridium*, *Enterococcus* and *Escherichia* were examined. Cats with symptomatic salmonellosis had increased (*P* ≤ 0·05) faecal proportions of *Clostridium perfingens* (6 *v.* 1 % of sequences) and an unidentified species from *Escherichia* subgenera *Shigella* (0·5 *v.* < 0·1 % of sequences) (data not shown).

**Table 1.** Potentially pathogenic bacteria detected by PCR and culture methods in the diet and faeces of domestic cats with (*n* 3) and without (*n* 9) symptoms of infection (expressed as number of samples)

			Faecal samples	
Item	Test result	Diet*	No symptoms	Symptomatic†
*Salmonella* detection PCR				
	Negative	2	9	0
	Positive	0	0	3
*Campylobacter* detection PCR				
	Negative	2	9	3
	Positive	0	0	0
Aerobic culture				
*Clostridium perfringens*				
	None reported	2	0	0
	Heavy	0	7	2
	Very heavy	0	2	1
*Clostridium* species				
	None reported	2	0	2
	Heavy	0	4	1
	Very heavy	0	5	0
Anaerobic culture				
*Escherichia coli*				
	Few	0	1	0
	Moderate	2	5	1
	Heavy	0	3	1
	Very heavy	0	0	1
*Enterococcus* species				
	None reported	0	0	1
	Moderate	1	0	1
	Heavy	1	5	1
	Very heavy	0	4	0

*Whole and ground 1–3-d-old chicks (Rodent Pro).

†Symptomatic = anorexia (*n* 1); diarrhoea (*n* 2).

**Table 2.** Predominant bacterial genera (expressed as percentage of sequences) in faeces of domestic cats with (*n* 3) and without (*n* 9) symptoms of infection

Phylum	Family	Genus	*Salmonella* negative	*Salmonella* positive	sem	*P* value
Actinobacteria	Coriobacteriaceae	*Collinsella*	5·1	3·5	1·6	0·49
		*Slackia*	0·2	0·1	0·1	0·65
Firmicutes	Clostridiaceae	*Clostridium*	15·8	22·3	3·1	0·18
	Enterococcaeae	*Enterococcus*	1·4	1·6	0·7	0·74
	Erysipelotrichaceae	*Allobaculum*	1·2	1·4	0·8	0·83
		Unidentified genera	0·7	0·2	0·3	0·19
	Eubacteriaceae	*Eubacterium*	4·2	3·1	1·7	0·38
	Lachnospiraceae	*Blautia*	10·1	12	2·8	0·65
		*Coprococcus*	0·4	5·6	1·4	< 0·01
		*Moryella*	0·6	0·1	0·3	0·27
		*Psuedobutyrivibrio*	3·6	4·3	0·9	0·50
		*Roseburia*	1·0	0·7	0·5	0·67
		Unidentified genera	16·6	12·3	2·4	0·22
	Oscillospiraceae	*Oscillibacter*	0·4	0·1	0·1	0·06
	Peptococcaeae	*Peptococcus*	10·3	10·9	2·5	0·87
	Peptostreptococcaeae	*Peptostreptococcus*	1·6	0·0	1·4	0·39
		Unidentified genera	0·3	0·4	0·1	0·35
	Ruminococcaceae	*Anaerotruncus*	2·3	1·4	0·4	0·09
		*Ruminococcus*	4·5	4·8	1·6	0·89
		Unidentified genera	2·5	1·1	0·8	0·20
Fusobacteria	Fusobacteriaceae	*Fusobacterium*	8·6	7·4	2·1	0·71
Proteobacteria	Campylobacteraceae	*Campylobacter*	1·6	0·3	1·7	0·61
	Enterobacteriaceae	*Escherichia Shigella*	0·3	1·1	0·3	0·03
		*Shigella*	1·8	3·5	1·3	0·41
	Succinivibrionaceae	*Anaerobiospirillum*	0·8	1·3	0·8	0·73
	Sutterellaceae	*Sutterella*	1·1	0·7	0·5	0·58

## Discussion

The gut of cats are colonised by trillions of bacteria, which exist in a symbiotic relationship with the host^(^^19^^)^. The majority of these bacteria are neutral to the host, whereas others confer benefits to the host, including proper development of the immune system, the digestion of food and absorption of nutrients, the production of key vitamins (e.g. vitamin K and biotin) and protection against invading pathogenic organisms. During infection, pathogenic bacteria compete with and displace the commensal species resulting in gastrointestinal upset, including inflammation, nausea, gas production and bloating, and diarrhoea^(^^15^^)^. Herein we identified alterations in the faecal bacterial genera of cats with symptomatic salmonellosis.

*Salmonella* are rod-shaped Gram-negative enterobacteria. Reported *Salmonella* isolates from cats with pathogenic infection include *S. typhimurium* (most commonly isolated), *S. cholerasuis*, *S. dublin*, *S. newport*, *S. arizonae*, *S. saintpaul*, *S. krefeld*, *S. typhisuis*, *S. enteriditis*, *S. hadar*, *S. manhattan*, *S. infantis* and *S. virchow*^(^^14^^)^. *Salmonella* infection in cats can be asymptomatic, or cause gastroenteritis, and/or septicaemia^(^^14^^)^. The symptoms noted herein, anorexia and diarrhoea, are typical of salmonellosis. Additional symptoms (not observed herein) include fever, lethargy, vomiting, weight loss and dehydration among others. Cats herein were monitored closely, the infections were self-limiting and resolved without treatment within a few days.

Exposure to pathogenic species of strains by faecal-to-oral transmission, and ingestion of contaminated feed may lead to zoonotic infection (transmissible between species). However, not all host animal species will develop infection or overt symptoms when a pathogenic species of strain is present (i.e. poultry species can act as a reservoir for pathogenic *Salmonella* species without overt signs of infection)^(^^20^^)^. Although the diet samples tested herein were negative for *Salmonella*, Kerr *et al.*^(^^10^^)^ detected *Salmonella* in 1–3-d-old chicks obtained from the same supplier. Whole-prey diet items containing gut contents make it inevitable that the animals ingesting them will be exposed to foreign bacteria. Contamination of poultry species with potentially pathogenic bacteria may occur pre-harvest (e.g. breeding, growth, etc.), during harvest (handling and transport) or after harvest (during slaughter, storage or transport). Because whole prey are typically fed raw, and freezing does not kill *Salmonella*, extra caution should be taken during production processes to reduce contact of whole prey with pathogenic bacteria.

The natural history, digestive physiology (e.g. rapid transit time) and commensal microbiota of healthy adult cats may allow them to tolerate exposure and harbour pathogenic bacteria without overt symptoms of disease^(^^14^^)^. However, exposure to pathogenic bacteria should be minimised in cats, and the risk for human subjects may be a concern. Proper handling of raw animal products, bowls and other surfaces that come into contact with the raw items can minimise the risk of exposure. Because cats themselves can harbour pathogens, such as *Salmonella*, without overt signs of infection, handling animals fed whole prey may increase risk of infection for human subjects. These potential risks mean that whole-prey feeding may not be appropriate for all households (e.g. people or animals that are immune-compromised).

*Salmonella* was not confirmed utilising the pyrosequencing technique in this study. However, the proportions of select faecal genera were modified in cats with symptomatic salmonellosis. These data indicate that pyrosequencing may not be a sensitive enough assay for detecting salmonellosis. Even though the culture techniques gave similar results for symptomatic and asymptomatic cats, salmonellosis may have been secondary to infection by another pathogenic bacteria. Both the results of the aerobic and anaerobic cultures (detection of other potentially pathogenic bacteria) and pyrosequencing (increased proportions of other potentially pathogenic bacteria) support the role of other pathogenic bacteria (e.g. *Clostridium perfringens* and *Escherichia coli* species) in the progression of gastroenteritis in these cats. We do not have enough data to determine which potentially pathogenic bacteria were responsible for the initial infection. Further investigations to determine the relationship between clinical status and microbial shifts may provide insight into the diagnosis of salmonellosis.
